# Imported malaria including HIV and pregnant woman risk groups: overview of the case of a Spanish city 2004–2014

**DOI:** 10.1186/s12936-015-0891-0

**Published:** 2015-09-17

**Authors:** María Fernández López, Jose Manuel Ruiz Giardín, Juan Víctor San Martín López, Jerónimo Jaquetti, Isabel García Arata, Carolina Jiménez Navarro, Noemi Cabello Clotet

**Affiliations:** Department of Internal Medicine, Fuenlabrada University Hospital, Fuenlabrada, C/Camino del Molino, 2, Zip Code 28942 Fuenlabrada, Madrid, Spain; Rey Juan Carlos University, Alcorcón, Madrid, Spain; Department of Microbiology, Fuenlabrada Universtiy Hospital, Fuenlabrada, Madrid, Spain; Doctors Without Borders, Madrid, Spain; San Carlos Clinical Hospital, Madrid, Spain

**Keywords:** HIV, Inmigrants, Malaria, Pregnancy, Public health, VFRs

## Abstract

**Background:**

Arrival of inmigrants from malaria endemic areas has led to a emergence of cases of this parasitic disease in Spain. The objective of this study was to analyse the high incidence rate of imported malaria in Fuenlabrada, a city in the south of Madrid, together with the frequent the lack of chemoprophylaxis, for the period between 2004 and 2014. Both pregnant women and HIV risk groups have been considered.

**Methods:**

Retrospective descriptive study of laboratory-confirmed malaria at the Fuenlabrada University Hospital, in Madrid, during a 10-year period (2004–2014). These data were obtained reviewing medical histories of the cases. Relevant epidemiological, clinical and laboratory results were analysed, with focus on the following risk groups: pregnant women and individuals with HIV.

**Results:**

A total of 185 cases were diagnosed (90.3 % *Plasmodium falciparum*). The annual incidence rate was 11.9/100,000 inhabitants/year. The average age was 30.8 years (SD: 14.3). Infections originating in sub-Saharan Africa comprised the 97.6 % of the cases. A total of 85.9 % were Visiting Friends and Relatives. Only a 4.3 % completed adequate prophylaxis. A total of 14.28 % of the fertile women were pregnant, and 8 cases (4.3 %) had HIV. None of them in these special groups completed prophylaxis.

**Conclusions:**

The incidence rate in Fuenlabrada is higher than in the rest of Spain, due to the large number of immigrants from endemic areas living in the municipality. However, the results are not representative of all the country. It seems to be reasonable to implement prevention and pre-travel assessment programs to increase chemoprophylaxis. Pregnancy tests and HIV serology should be completed for all patients to improve prophylactic methods.

## Background

Malaria is the most common and lethal parasitic disease in the world and one of the most important public health problems. According to data from the World Health Organization (WHO), malaria is endemic in 104 countries, which suggests that half of the world’s population is at risk of suffering from this disease. The WHO estimates that 198 million cases of malaria occurred worldwide in 2013 and the disease led to 584,000 deaths. The worst scenery can be found in the WHO African Region, where approximately a 90 % of all of the malaria deaths occur; children under 5 years in this area account for a 78 % of all the deaths [1].

Spain has 46.5 million inhabitants [2] and is defined as “the port of entry to Europe from Africa”. The large number of immigrants arriving in the country from malaria endemic areas has led to a rise of this parasitic disease cases in the last years.

The last reported autochthonous case of malaria in Spain was in 1961, while malaria was officially declared eradicated in 1964. In October 2010, one case of autochthonous malaria due to *Plasmodium vivax* was diagnosed in Aragon, north-eastern Spain. However, and due to the presence of the vector *Anopheles atroparvus* in Spain, autochthonous malaria is not an unexpected fact [3]. In addition, sporadic autochthonous transmission of vector-borne diseases in continental Europe is possible; this fact could be appreciated after the occurrence of several emerging vector-borne disease outbreaks in different countries in Europe [4].

In 2012, according to data from the National Network of Epidemiological Vigilance of the National Centre of Epidemiology (Red Nacional de Vigilancia Epidemiológica del Centro Nacional de Epidemiología), 484 cases were reported to the WHO, all of which were imported cases, been caused mainly by *Plasmodium falciparum* [5]. In 2013, the incidence rate in Spain was 1.25 cases/100,000 inhabitants [6].

In Spain, most of the imported malaria cases affect immigrants who live in Spain and travel to their home countries to visit their friends and relatives (Visiting Friends and Relatives, VFR). This risk group represents an important percentage of the imported malaria cases described in most of the studies about this matter [7–9]. In these cases, the high risk of malaria can be attributed to various factors. In one hand, they do not receive pre-travel assessment. On the other, there is a low perception of risk due to a sense of immunity against infection. And finally, there are cultural and/or language barriers. In addition, sometimes the travel is not planned, with people having to travel rapidly to their home countries to attend to a specific event [10]. Usually, they travel to rural areas and stay there for long periods of time [10, 11]. Traveller Visiting Friends and Relatives, who are descendents of immigrants born in a non-endemic zone, represent a special population [12]. Most of them are children who travel to the home countries of their parents and who do not possess natural semi-immunity against malaria [7, 10, 11, 13]. In fact, they sometimes suffer severe malaria.

The Community of Madrid is in the centre of Spain. It has almost 6.5 million inhabitants and a 14.28 % of immigrant population (930,366 foreign inhabitants are registered). Romanians are the most numerous (22.6 %), followed by Moroccans (9 %) and other African nationalities (3 %) [14]. Malaria has been a mandatory reporting disease in Madrid since 1995, when the responsibility of the surveillance programmes in Spain was transferred from the national govern to the regional governs [15]. However, it has always been a mandatory reporting disease in Spain. Between 2004 and 2013, 1214 cases of malaria were reported (between 350 and 400 cases a year), although due to under-reporting, the total number of imported cases is thought to be higher. In 2013, 143 cases were reported, with an accumulated incidence rate of 2.24 cases/100,000 inhabitants [6] (considering in the denominator the registered population of Madrid from 2013 census). In 2014, between January and June, 53 cases of malaria were reported [16].

Regarding the municipality of Fuenlabrada, it has a population of 200,312 inhabitants living in 39.21 km^2^, with a 13.6 % of immigrants (January, 2015) [17]. It is one of the regions of the community of Madrid with the highest number of reported cases of malaria, due to the fact that a large number of immigrants from endemic areas have settled in here [18]. This city, in the south of Madrid, is where the presented study was focused.

## Objective

The main objectives of this study are to evaluate the high incidence rate of this parasitic disease in this Spanish city (a non-endemic area), together with the description of the sociodemographic, epidemiological and clinical characteristics of the cases of malaria diagnosed in the Fuenlabrada University Hospital for a 10 years period (from its opening in June 2004 until June 2014). In addition, the lack of chemoprophylaxis is also analysed. Two risk groups are taken into account in the study: pregnant women and Human Immunodeficiency Virus (HIV).

## Methods

As it has been already described in the Introduction section, the study is focused on the municipality of Fuenlabrada. The malaria cases under analysis were all of those diagnosed between June 2004 and June 2014 in the Fuenlabrada University Hospital. This centre attends the whole population of the city, ant it has 350 beds.

In the municipality of Fuenlabrada, there were a total of 27,187 registered foreigners (13.6 %), with the following distribution: Romanian (22.5 %), Moroccan (16.2 %), Nigerian (8.9 %), Chinese (7 %), Colombian (4.6 %), and Equatoguinean (4.4 %) [17]. The Sub-Saharan African population registered in Fuenlabrada is 4377 inhabitants. Approximately a 14 % of the Sub-Saharan population in the Community of Madrid lives in the municipality of Fuenlabrada [14]. It is important to emphasize that many foreigners are not legally registered and this number is higher within the Sub-Saharan population.

The study was performed as a descriptive retrospective survey performed in the Fuenlabrada University Hospital. The clinical history numbers of the cases were provided by the Departments of Microbiology and Preventive Medicine. The database was completed and designed in a retrospective manner after reviewing the clinical history of the cases. The usage of these clinical histories was approved by the Ethics Commission of the Fuenlabrada University Hospital.

The main analyzed data were the following: sociodemographic (age, sex, country of origin and residence, “visiting friends and relatives”), epidemiologic (country of the stay, duration of stay, month and year of diagnosis, time in Spain before diagnosis, time between the onset of symptoms and diagnosis), clinical (symptoms and clinical signs -presence of splenomegaly, complications), laboratory results (PCR, haemoglobin, platelets and white blood cells, including *Plasmodium* species and parasitaemia), type of treatment, days of hospital stay in case of admittance, intake or not of prophylaxis, deaths and analysis of characteristics of special groups: pregnancy and VIH.

Malaria diagnosis was confirmed in 168 cases (90.9 %) by microscopy on peripheral blood smear or thick blood smear. The diagnosis was completed exclusively by genomic amplification (PCR) in six cases (3.2 %), only by antigenaemia (Ag) in four (2.2 %), and by a combination of Ag and PCR in seven cases. The genomic amplification tests were performed on the National Centre of Microbiology of the Spanish Ministry of Science and Innovation in the city of Majadahonda. In the case of pregnant, pregnancy tests were performed on 26 women (43.3 %) out of the 60 who were of reproductive age (between 16 and 50 years). Nine positive cases of pregnancy (14.28 %) were found. Regarding HIV, a serological test was completed in 89 cases (48.1 %), confirming HIV in eight patients (8.9 %), with three false positives (3.3 %).

A descriptive analysis was performed. Qualitative values are presented using percentages. Mean and standard deviation (SD) or median and interquartile range (IQR), when the continuous variable did not follow a normal distribution, have been used for quantitative values. Corresponding non-parametric tests were used to compare quantitative variables. Incidence rates were calculated per 100,000 inhabitants/year. Following the approach of the National Network of Epidemiological Vigilance of the National Center of Epidemiology [18], the denominator is the total population at risk (taken as the one obtained from the city census of 2013) [17], while the numerator is the total number of cases reported to the centre in that year. Recently arrived immigrants have not been taken into account for the incidence rate calculation.

Data have been processed using SPSS 15 software.

## Definitions

*Imported case* case acquired in a zone where malaria is endemic and diagnosed in a non-endemic area by microbiological criteria (i.e. peripheral blood smear, thick blood smear, positive antigen or genomic amplification).

*Sub*-*Saharan Africa* all African countries that are fully or partially located south of the Sahara (excluding Sudan, even though Sudan sits in the Eastern portion of the Sahara desert) (Political definition of “Major regions”).

*VFR* “Visiting Friends and Relatives”: immigrants who travel to their home countries to visit their friends and relatives [10].

*Immigrant VFR* immigrant who travels to his home country to visit friends and family once he is established in his country of residence [12].

*Traveller VFR* a descendent of immigrants, born in a non-endemic zone, who travels to his parents’ home country to visit friends and relatives [12].

*Immigrant* a foreigner who has recently arrived in Spain (within the last year) with no subsequent travel to his/her country [8].

*Traveller* a person born in Spain who travels to an endemic zone [10].

*Parasitological cure* peripheral blood smear, antigenaemia or thick blood smear after completing antimalarial treatment.

## Results

From the opening of Fuenlabrada University Hospital in June 2004 until June 30, 2014, a total of 185 cases of malaria have been diagnosed. All of them were imported.

The resulting annual incidence rate was 11.9/100,000 inhabitants/year in 2013.

The average number of reported malaria cases was 18.5 cases/year, with the highest number of cases (25) in 2012. The month with the highest number of diagnoses was September, with a total of 31 cases (16.8 %).

With respect to nationalities, 87.6 % (162) were born in Sub-Saharan Africa, mainly Nigeria (42.7 %) and Equatorial Guinea (39.5 %). Of the rest, 20 (10.8 %) were born in Spain but were children of immigrants. The remainder consisted of one from Portugal, one from Brazil, and one non-VFR from Spain. Of the affected cases, 97.6 % were infected in sub-Saharan Africa, 45.9 % (85) in Nigeria, 45.4 % (84) in Equatorial Guinea, 3.2 % (6) in Guinea Conakry and 1.1 % (2) in Cameroon. There was one case reported in each of the following: Ivory Coast, Congo, Angola, Brazil, Senegal and Vietnam. Two cases had unknown origins. The median stay in the endemic zone was 30 days (IQR: 21–60). The median time in Spain before diagnosis was 9 days (IQR: 5–15).

A total of 85.9 % (159) of infections were VFR: 141 were immigrant VFR and 18 were traveller VFR. Immigrants comprised 13.5 % (25 cases/185) of infections. Only one subject was a traveller (a young Spanish boy who travelled to Vietnam).

The median time from symptoms to diagnosis was 4 days (IQR: 2.5–7) and the most common symptom was fever in 95.7 % (177) of the cases, followed by digestive symptoms in 50.8 % (94) and headache in 48.1 % (89). In 29 patients (15.7 %), splenomegaly was responsible for the presentation. 1 patient presented a spontaneous rupture of the spleen. There was one severe case in a woman that presented a sickle cell crisis with bilateral amaurosis caused by an obstruction of the central retinal artery.

A total of 176 patients (95.1 %) required hospital admission, with a median admission of 3 days (IQR: 2–5). 8 patients (4.3 %) required admittance to the ICU, following the WHO clinical criteria for severe malaria (impaired consciousness, prostration, multiple convulsions, deep breathing and respiratory distress, acute pulmonary edema and respiratory distress, circulatory collapse or shock, acute kidney injury; clinical jaundice plus evidence of other vital organ dysfunction; and abnormal bleeding) [19].

The most common laboratory finding was high levels of C-reactive protein (CRP) in 145 of the tested subjects. The most common haematological alteration was thrombocytopaenia (values under 150,000 platelets/ml) in 77.3 % (143) of the cases [median of 107,000 (IQR: 73,500–139,000)]. 51.9 % (96) presented anaemia (haemoglobin under 12 g/dl in women and 13 g/dl in men), with an average of 12.35 g/dl (SD: 2.15). There were no alterations in the white blood cell series: 33 (17.8 %) presented leukopaenia (levels under 3500 leukocytes/µl). Percentage parasitaemia at diagnosis was obtained in 160 cases (86.8 %), with a range between <1 and 20 % and an average of 0.9 % (IQR: 0.9–2).

Regarding the type of *Plasmodium*, 90.3 % (167) of cases were *P. falciparum,* 3.2 % (6) were mixed parasitism (*P. falciparum* + *P. vivax/Plasmodium ovale*), 1.6 % (3) were *P. ovale,* 1.6 % (3) were *P. vivax*, and 1.1 % (2) were *Plasmodium malariae*. In 2.2 % (4) of the cases, the species of *Plasmodium* was not identified.

A total of 29 patients (15.7 %) took chemoprophylaxis. Of these, eight patients (4.3 %) did it correctly. By origin, only 3 (4.1 %) of those born in Equatorial Guinea (73 patients) and 1 (1.2 %) of the Nigerians (79 patients), completed prophylaxis correctly.

Treatment was received in 182 cases (98.3 %). A 84.4 % (156) received treatment with combinations of quinine or derivatives (hydroxychloroquine, mefloquine or quinine sulfate), a 12.4 % (23) with atovaquone–proguanil, and a 1.6 % (3) with artemisinin. Of those treated with atovaquone–proguanil, 7 (30.4 %) were treated with derivatives of quinine at diagnosis, but treatment was modified due to an adverse effect of quinine.

No deaths resulted.

### Malaria and pregnancy

The most common symptom was fever, present in seven cases (77.8 %). Characteristically, almost all of the pregnant women presented anaemia (8 cases, 88.9 %), with average haemoglobin values of 10.1 g/dl (SD: 1.72) compared to the 11.45 g/dl (SD: 1.88) in non-pregnant women of reproductive age (*p* = 0.06). 6 cases (66.7 %) received quinine and clindamycin, 2 (22.2 %) received artesunate and the rest received quinine and doxycycline due to previous miscarriage. None completed proper prophylaxis (Table 1).

**Table 1:** Cases of malaria and pregnancy

	Patient 1	Patient 2	Patient 3	Patient 4	Patient 5	Patient 6	Patient 7	Patient 8	Patient 9
Age	30	31	32	31	35	36	33	32	26
Origin^a^	RD Congo Immigrant	Nigeria VFR	Nigeria VFR	Equatorial Guinea VFR	Nigeria VFR	Equatorial Guinea VFR	Nigeria VFR	Equatorial Guinea VFR	Equatorial Guinea VFR
Gestation Trimester^b^	2°	1°	3°	2°	1°	3°	3°	2°	3°
Parasitism^c^	<1 %	5 %	<1 %	<1 %	1.40 %	<1 %	1 %	<1 %	<1 %
Evolution	Lost	Recovery	Recovery	Recovery	Recovery	Recovery. Caesarean	Recovery	Relapse due to incomplete treatment. Recovery	Recovery
New-born and weight		Healthy 3.11 kg.	Healthy 2.88 kg	Healthy 2.95 kg		Foetal distress 3.46 kg	Healthy 2.76 kg	Healthy 3.93 kg	Healthy 3.18 kg
Complication		No	HIV A2	HIV A2	Miscarriage	None No trophozoites in a placental study	Anaemia Hb: 6 g/dl	No	No

^a^All VFR are immigrants VFR

^b^All of the pregnant women were multiparous

^c^All cases were *Plasmodium falciparum*

### Malaria and HIV

All the cases were VFR-immigrants. None had completed chemoprophylaxis (Table 2). Four were diagnosed with HIV at the time malaria was diagnosed, with an average CD4 of 221/mm^3^ (IQR: 51–325). The other four had known HIV [with an average CD4 count at the time of the malaria diagnosis of 394/mm^3^ (IQR: 239–405)]. There was a difference between both groups, though it was not statistically significant (*p* = 0.29).

**Table 2:** Cases of malaria and HIV

	Patient 1	Patient 2	Patient 3	Patient 4	Patient 5	Patient 6	Patient 7	Patient 8
Age/sex	41/Male	36/Female	23/Female	32/Pregnant female	31/Pregnant female	33/Female	33/Female	31/Female
Origin	Guinea Conakry	Equatorial Guinea	Nigeria	Nigeria	Equatorial Guinea	Equatorial Guinea	Equatorial Guinea	Nigeria
HIV infection^a^	Diagnosed	Diagnosed	Known HIV	Known HIV	Known HIV	Diagnosed	Diagnosed	Known HIV
Parasitism	8 %	<1 %	15 %	<1 %	<1 %	<1 %	2 %	<1 %
HIV State	HIV A3	HIV A2	HIV C3	HIV A2	HIV A2	HIV A3	HIV A2	Unknown
Evolution	Cerebral malaria. Admittance to ICU. Recovery	Diagnosis of malaria and HIV during an anaemia study	Recovery	Recovery. Healthy new-born	Resuming (HAART). Healthy new-born	Recovery.	Haemoglobin <8 g/dl. Required transfusion. Recovery	Moved to her reference centre

Diagnosed: coinciding with malaria diagnosis

Known: HIV diagnosis before diagnosis of malaria

^a^Moment of HIV infection

In recently diagnosed cases of HIV, the average levels of haemoglobin were haemoglobin: 9.43 g/l (SD: 3.1) and the median levels of platelets were 92,500 (IQR: 74,500–170,500), both under the levels found in the cases with previously known HIV [haemoglobin: 10.8 g/dl (SD: 1.7) and 172,500 (IQR: 110,000–227,500), respectively]. The differences were not significant (*p* = 0.10).

## Discussion

This study described all cases of imported malaria in Fuenlabrada (Madrid, Spain) during the period 2004–2014. The most remarkable finding is the high incidence rate, which far exceeds those from the Community of Madrid and from the rest of Spain. In addition, it was also observed that most of the diagnosed malaria cases affect sub-Saharan patients, and that there is a high percentage of VFR. They usually attended the hospital quite quickly and did not complete prophylaxis. A significant percentage are cases within pregnant and HIV+ risk groups, which present a higher risk of severe malaria.

Regarding the very high incidence rate observed in the studied area (11.9/100,000 inhabitants/year in 2013), it has been tried to obtain the most reliable values by excluding from it those cases of immigrants who have recently arrived in Spain (less than 1 year). These are usually immigrants following routes to other European countries, who stay for a short time in Spain visiting friends and relatives and preparing their trip. It is in these moments when malaria symptoms appear (mostly fever), and they go to hospital. These cases are then notified, but in many of them, there is not a clinical following up, fact that shows up when medical histories are analysed.

One of the possible causes of the difference between the obtained results and the incidence rates of the community of Madrid and the rest of Spain, may be an excess of diagnostic tests in the area under analysis, where the sub-Saharan population is very important and malaria quite frequent. In fact, in the emergency department, malaria is tested in all cases with fever coming from Nigeria or Equatorial Guinea, disregarding their time of permanence in Spain (sometimes travelling details are hidden by patients). This may not be the case in other areas, where some cases of malaria are not diagnosed due to the absence of suspicion and the corresponding tests. For example in Leganés [8], another city in the South Ring of Madrid, the approximate incidence rate is around the 8 % in 2008 (15 cases; 184,209 inhabitants), which although is lower than the one found in the study in Fuenlabrada, it is still a quite high rate.

Regarding the population under study, no reliable data are available apart from those of the 2013 census, and so only registered citizens are included for the computation of the incidence rate (denominator). Of course, there is a percentage of the immigrant population which is in an extreme irregular situation, that does not allow them to even register or to get a sanitary card, but they can still be treated for free in the emergency services. These cases have been included in the numerator but, as it has been explained, not in the denominator. However, taking into account that immigrants can get registered in the census in Spain with no need of having a residence permit, the difference in incidence rate due to the non-consideration of these “extreme irregular” cases in the total population does not have a significant effect on the rate calculation.

The population coming from the Sub-Saharan Africa, is mainly distributed in two areas of the community of Madrid (Madrid centre and the South Ring), with a 74.52 % of the immigrants and they tend to stay in districts by nationalities. In Fuenlabrada (within the South Ring), a 55.5 % of sub-Saharan immigrants come from Nigeria and a 27.7 % from Equatorial Guinea. In the rest of the community of Madrid, a 29.78 % come from Nigeria and a 19.39 % from Equatorial-Guinea [14]. This population pattern confers a special characteristic to the area of Fuenlabrada, which differs from other Spanish health areas.

The majority of the cases of imported malaria are produced by *P. falciparum*, which is more frequent in Nigeria and Equatorial Guinea (a former Spanish colony); this pattern was also observed in other case series in Europe [10, 20] and Spain [7–9, 11]. Other countries as UK or Australia, have a higher number of immigrants coming from South-Asia; there *P. vivax* is the prevalent species, and it is present in more than a 50 % of the diagnosed cases [8]. A high percentage of VFR (almost 86 %) has been observed, like in the majority of studies regarding imported malaria [7, 8]. The population traveller-VFR, who was not born in an endemic area, has to be highlighted. Only one subject was a traveller; the absence of cases in domestic travellers could be due to the current economical situation. This area in the south of Madrid is a middle- and working-class area, which could explain the reduction in leisure travel to tropical zones [8] or the completion of proper prophylaxis.

The low number of days until diagnosis (3 days) of Equatorial Guinean population compared to the average of 4.5 days for Nigerians VFR, could be explained due to the absence of a language barrier for the former (mother tongue is Spanish), who attend the hospital sooner. The median time from symptoms to diagnosis was 4 days (IQR: 2.5–7) similar to another study published in southern Madrid [8]. In a study in the city of Murcia (Spain), the median was 7 days (with a range of 1–30 days) [9]. Regarding this fact, in some cases patients who have already suffered malaria and who know the symptoms, once back in Spain and after the appearance of fever, quickly attend the hospital to receive treatment. This is one of the reasons of the low number of days between the onset of symptoms and diagnosis.

Regarding the high number of cases who required hospital admission (95.1 %), it is explained due to the fact that it is common practice in Fuenlabrada Universtiy Hospital to hospitalize nearly all malaria cases to check their correct evolution with the treatment, to carry out analytical vigilance (anaemia) and to follow up and make a thick blood smear after 48 h. The infrequent use of chemoprophylaxis is a well-known issue. In the presented study, the rate of properly completed prophylaxis was 4 %, similar to previous rates that range from 3 to 15 % [9, 11, 13, 21]. The main reason for this fact is the ignorance of the need of chemoprophylaxis. The language and cultural barrier usually makes getting pre-travel counselling in Hospital, Primary Care Centre or in NGO difficult or even impossible. Some of the cases, the “extreme irregular” ones, do not have access to an International Vaccination Centre to get chemoprophylaxis.

It was observed that travellers-VFR (born in Spain) completed prophylaxis more frequently (16.7 %) than immigrants VFR (born in endemic area) (2.8 %). The explanation to this result could be that parents could have gone to the Primary Care Centre with their children (for a routine revision). There the paediatrician gives some pre-travel counselling about chemoprophylaxis and others, which is followed by the children (under the supervision of their parents), but not by the parents themselves (who were born outside of Spain and who have a misperception of the possible risk).

In Fuenlabrada University Hospital, after the experience with such a large number of cases, pre-travel counselling is added in the clinical report and the necessary prescription is given to the patients, as a way of increasing the correct use of chemoprophylaxis.

### Malaria and pregnancy

Malaria in pregnancy is associated with increases in maternal, fetal, and perinatal morbidity and mortality [22], and also with increased risks of severe anaemia, maternal death, miscarriage, premature labour, low birth weight and perinatal death.

The characteristics of the cases are similar to a previous study [22]. Weights less than 3 kg were observed in cases of pregnant women with HIV and in the patient with severe anaemia we can see in [23]. Two of the pregnant women were asymptomatic, but both presented moderate anaemia and parasitaemia under 1 %; with regards to this finding, one study in Mozambique observed that anaemia was the only sign associated with submicroscopic parasitaemia [24].

Given the high risk of maternal-fetal complications and the frequent occurrence of anaemia in such patients, the recommendation that a pregnancy test be completed in all cases of malaria in women of reproductive age (and of completing parasitological studies on all pregnant women who originate from an endemic zone, even if they are asymptomatic and only present anaemia), could be valuable.

### Malaria and HIV Infection

Among the infections that affect the human population, malaria and HIV/AIDS are responsible of most of the deaths. The U.S. Centers for Disease Control and Prevention (CDC) consider malaria as an opportunistic infection in patients with HIV in areas with geographic overlap [25]. Considerable overlap exists between symptoms of malaria, acute HIV infection and other opportunistic infections related to HIV, with the cardinal symptom of all those situations being fever [26]. Following up the recommendations from CDC a screening should be performed unless the prevalence of undiagnosed HIV infection has been documented as under 0.1 % [27].

Regarding the large number of positive HIV cases (8.9 %), although this rate is very high, it could be higher if a HIV serological test was completed in all cases of imported malaria. One of the reasons of these values may be the high HIV incidence in Sub-Saharan Africa (which is the highest worldwide). These tests are done because given the prevalence of HIV in sub-Saharan Africa and the large population of this precedence in our area, HIV detection tests should be completed as a public health measure.

The possibility of false positives for HIV in patients with malaria cannot be overlooked (three patients in our study) [28].

Is it important to evaluate that none of the patients had completed previous chemoprophylaxis. In a French study on imported malaria that described 190 cases of imported malaria in patients with HIV, 10.5 % had correctly completed prophylaxis [29]. In a study in the community of Madrid [30], only two of 32 patients with HIV-malaria co-infection had taken a correct form of chemoprophylaxis. As in the present study, all of the patients were semi-immune.

In cases of imported malaria, it has been observed that there is a higher risk of severe malaria almost exclusively in patients with HIV infection and CD4 counts under 350/mm^3^ [29]. In this study, the average lymphocyte CD4 count at diagnosis was 306/mm^3^, similar to the French study, with an average CD4 count at diagnosis of 299/mm^3^. It should be highlighted that no studies have been found that compare cases of imported malaria with concomitant diagnoses of HIV and malaria. In the presented study (even with the scarce number of cases), such cases exhibited greater immunosuppression (average CD4 count of 221/mm^3^). The severely affected patient with HIV infection exhibited a CD4 count of 10/mm^3^ (the lowest level in the entire series). The scarce number of patients with HIV may explain why there were no significant differences in any of the data analysed.

## Conclusions

From this study it can be concluded that the incidence rate of imported malaria in Fuenlabrada is higher than in the rest of Spain, due to the large number of immigrants from endemic areas living in this municipality. However and due to the special distribution of the population of the city, the results are not representative of all Spain or the community of Madrid. Most of the diagnosed malaria cases affect sub-Saharan patients and are due to *P. falciparum*. There is a high percentage of VFR and most of them did not complete prophylaxis. The population traveller-VFR, who was not born in an endemic area, has to be highlighted. It seems to be reasonable to implement prevention and pre-travel assessment programmes, especially in VFR, to increase the number of people who correctly perform chemoprophylaxis. Regarding pregnant woman risk group, it can be recommended that a pregnancy test is completed in all cases of malaria in women of reproductive age. In the case of the HIV risk group it can be concluded that HIV detection tests could be evaluated in all cases of imported malaria to improve prophylactic methods and as a public health measure.
