# Erratum to: Imported malaria including HIV and pregnant woman risk groups: overview of the case of a Spanish city 2004–2014

**DOI:** 10.1186/s12936-016-1216-7

**Published:** 2016-03-16

**Authors:** María Fernández López, Jose Manuel Ruiz Giardín, Juan Víctor San Martín López, Jerónimo Jaquetti, Isabel García Arata, Carolina Jiménez Navarro, Noemi Cabello Clotet

**Affiliations:** Department of Internal Medicine, Fuenlabrada University Hospital, Fuenlabrada, C/Camino del Molino, 2, 28942 Fuenlabrada, Madrid, Spain; Rey Juan Carlos University, Alcorcón, Madrid, Spain; Department of Microbiology, Fuenlabrada University Hospital, Fuenlabrada, Madrid, Spain; Doctors Without Borders, Madrid, Spain; San Carlos Clinical Hospital, Madrid, Spain

## Erratum to: Malar J (2015) 14:356 DOI 10.1186/s12936-015-0891-0

After publication of the original article [1], the authors noticed that an item of information had been omitted from the ‘Acknowledgements’ section. The section should have read:

“The present work has been financed by the Fuenlabrada University Hospital, Madrid, Spain. This study received financial support from the Red de Investigación Cooperativa en Enfermedades Tropicales (RICET-RD12/0018/0008), VI PN de I+D+I 2008–2011. ISCIII—Subdirección General de Redes y Centros de Investigación Cooperativa; y fondos FEDER.”

The phrase “y fondos FEDER” should have been included, to acknowledge the additional source of financial support for the article.
